# Insights from a combination of theoretical and experimental methods for probing the biomolecular interactions between human serum albumin and clomiphene

**DOI:** 10.1039/c8ra08237e

**Published:** 2018-12-05

**Authors:** Seyed Zachariah Moradi, Sajad Moradi, Amin Nowroozi, Komail Sadrjavadi, Negin Farhadian, Hosna Ehzari, Mohsen Shahlaei

**Affiliations:** Student Research Committee, Kermanshah University of Medical Sciences Kermanshah Iran; Nano Drug Delivery Research Center, Kermanshah University of Medical Sciences Kermanshah Iran; Pharmaceutical Sciences Research Center, School of Pharmacy, Kermanshah University of Medical Sciences Kermanshah Iran; Department of Medicinal Chemistry, Medical Biology Research Center, Faculty of Pharmacy, Kermanshah University of Medical Sciences 67346-67149 Kermanshah Iran mohsenshahlaei@yahoo.com mshahlaei@kums.ac.ir +98-831-34276493 +98-83-34276489

## Abstract

In this study, the interaction of clomiphene (CLO), a non-steroidal and ovulatory stimulant drug employed in the treatment of infertility, with human serum albumin (HSA), the most abundant plasma transport protein, was investigated using spectrofluorometric, FT-IR, UV-Vis, and molecular modeling methods. The obtained results indicated that the binding of CLO to HSA led to intense fluorescence quenching of HSA *via* a static quenching mechanism, and that the process of CLO binding to HSA was enthalpy driven. By using experimental and theoretical methods, it was confirmed that as a result of binding CLO, slight conformational changes in HSA occurred. Also, the negative Δ*H* of interaction indicated that the binding of CLO with HSA was mainly enthalpy driven. The experimental and computational results suggested that hydrogen bonds and van der Waals interactions played a major role in the binding, with overall binding constants of *K* = 3.67 × 10^9^ M^−1^ at 286 K and 6.52 × 10^5^ mol L^−1^ at 310 K. Moreover, the results of molecular modeling showed that Asp234, Phe228, Leu327, and Arg209 in HSA had the highest interaction energies with the ligand.

## Abstract 2

In this study, the interaction of clomiphene with human serum albumin (HSA), the most abundant plasma transport protein, was investigated using spectrofluorometric, FT-IR, UV-Vis, and molecular modeling methods.

## Graphical abstract

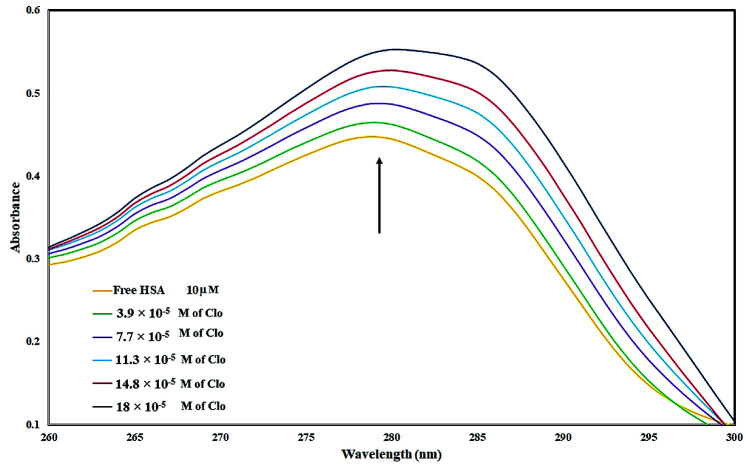

## 1. Introduction

**Scheme 1:**
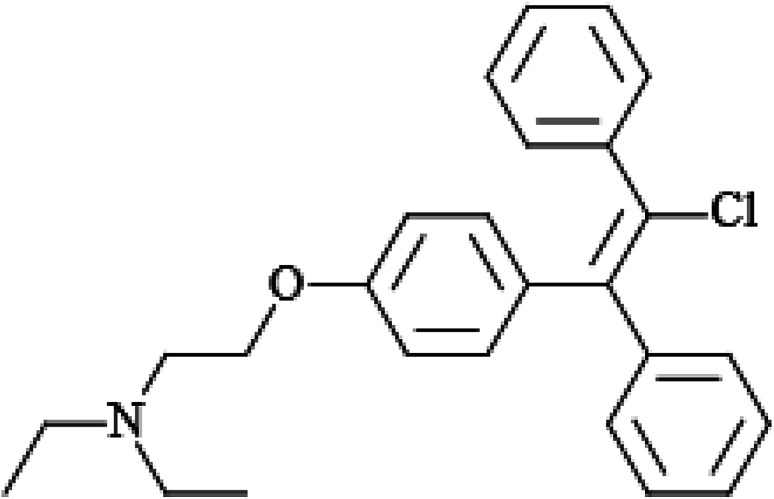
2D chemical structure of clomiphene (CLO).

Recently, the growing trend of delayed child-bearing in most industrialized countries has increased the demand for infertility treatment.^1–3^ This problem can be solved either by fertility drugs (such as clomiphene citrate) or aggressive assisted reproductive methods. Clomiphene (CLO), as a nonsteroidal triphenylethylene derivative, shows antagonist and estrogen agonist characteristics (Scheme 1).^4^ It is the first-choice treatment in the management of unexplained infertility and has been applied for the induction of ovulation. CLO acts by binding to the estrogen receptors in the pituitary gland and hypothalamus and also efficiently obstructing negative feedback from estrogen circulation, stimulating release of the follicle-stimulating and gonadotropin hormones. As a result of these changes, the follicle grows and ovulation occurs.^5,6^

Because CLO is a selective estrogen receptor modulator, it is used in estrogen receptor positive breast cancer prevention and treatment.^7^ Also, it is reported that CLO prohibits cellular proliferation of breast cancer cells.^8^ CLO exhibited antitumor activity in the early stage of a clinical trial during the treatment of advanced breast cancer.^9^

As soon as the drug enters the systemic circulation, the drug is exposed to blood proteins, *i.e.* human serum albumin (HSA), which is the major plasma protein in the blood. This exposure causes the drug to be divided into two populations: the free drug, and drug–HSA complexes. The free drug fraction has the most important biological/pharmaceutical effects and really only this fraction is distributed in the cells and biological fluids. The drug–HSA complex provides a supply for the free drug and also prolongs the duration of the drug's action.^10^ The binding of the drug to plasma HSA influences its delivery and efficacy: through holding the drug in the plasma and confining its clearance, there is an increase the pharmacokinetic half-life, however this complexation decreases the drug distribution within the tissues and can restrict the contact of the drug with the biological target.^11,12^ HSA is a non-glycosylated and single-chain protein comprising 585 amino acids, with a molecular weight of 66 500 Da.^13–15^ HSA has various physiological functions including contributing to colloid osmotic blood pressure and acting as a carrier, distributor and metabolizing agent for many metabolites and molecules such as drugs, fatty acids, hormones, cations and anions, and amino acids.^16^ It is known that the binding of drugs to HSA alters their free concentration, distribution and metabolism.^16^ In order to study the interactions between HSA and ligands, a variety of methods have been widely used and reported.^17–19^ Molecular dynamics (MD) simulation provides a detailed insight into protein–ligand complexes on the atomic scale.^19–21^ This computational method allows the observation of alterations in the protein backbone structure or side chains upon interaction with a ligand.^22,23^ Many studies have employed MD simulation for understanding the main protein–ligand binding interactions.^24–27^

In this study, a combination of electrochemical and spectroscopic methods was employed to comprehensively study the interactions between CLO and HSA. The conformational changes of the HSA as a result of binding to CLO have been further studied by the molecular docking and MD simulation methods.

## 2. Material and methods

### 2.1. Materials

HSA (fatty acid free) and CLO were purchased from Sigma-Aldrich and used without further purification. Other chemicals used in this study were of analytical grade and were used as received.

### 2.2. UV-Vis absorption spectral measurements

The UV spectra were acquired with an Agilent 8453 spectrophotometer with a 1.0 cm quartz cuvette (Waldbronn, Germany) using PBS buffer. During UV experiments, the HSA concentration was kept constant at 10^−5^ mol L^−1^ while the CLO concentration was varied from 3.9 × 10^−5^ to 18 × 10^−5^ mol L^−1^.

### 2.3. Steady-state fluorescence experiments

Fluorescence spectra were collected using a PerkinElmer LS 45 Fluorescence Spectrometer at an excitation wavelength of 290 nm. The emission spectra were recorded using a 1 cm path length quartz cuvette (3.0 ml) at wavelengths from 300 to 400 nm. The concentration of HSA was 1 × 10^−6^ M and CLO solution was added to HSA at various ratios (0.5 : 1 to 7 : 1) at 5 min time periods. Fluorescence measurements at the two temperatures of 286 and 310 K were obtained to probe the important binding forces and quenching mechanism of CLO–HSA. To investigate the conformational changes of HSA after addition of CLO, synchronous fluorescence spectroscopy (SFS) at different scanning intervals (Δ*λ* = 15, 60) was used. It must be noted that for the Δ*λ* = 15 nm measurements, the concentrations of HSA and CLO were 1 × 10^−6^ M and 4 × 10^−6^ to 2.73 × 10^−5^ M, respectively, and at Δ*λ* = 60 nm the concentrations of HSA and CLO were 1 × 10^−7^ M and 8 × 10^−7^ to 3.8 × 10^−6^ M, respectively.

### 2.4. FTIR experiments

All spectra were collected with a Shimadzu FT-IR spectrometer (Japan) *via* the ATR mode at a spectral resolution of 4 cm^−1^ with 64 scans using a KBr beam splitter. Initially, the spectra of CLO and buffer solutions were recorded to give the background signals. In the next step, the emission spectra of HSA in the absence and presence of increasing amounts of CLO in the buffer solution (molar ratios of 1 : 1, and 3 : 1) were recorded. Finally, each corresponding background spectrum was manually subtracted from the spectra of the CLO–HSA complex.

### 2.5. Electrochemical experiments

Electrochemical experiments were performed using a μ-Autolab TYPE III (Meterohm) equipped with NOVA software (Version 1.11). The three-electrode electrochemical testing system included a carbon paste electrode (CPE) modified by TiO_2_ as the working electrode, a Pt wire with a diameter of 0.5 mm as the counter electrode and an Ag/AgCl electrode as the reference electrode. At first, the electrolyte contained 5 × 10^−3^ mol L^−1^ K_3_Fe(CN)_6_/K_4_Fe(CN)_6_, and 10 × 10^−1^ mol L^−1^ KCl at pH = 7.4 was applied as a redox probe. Next, with a magnetic stirring device, 10 mL electrolyte was added to the electrochemical cell. Before collecting the experimental data, the electrodes were immersed in Tris–HCl solutions. In the following step, defined amounts of HSA were continuously added to the known amount of CLO (1 × 10^−4^ mol L^−1^). The electrochemical impedance spectroscopy (EIS) measurements were carried out within the frequency range of 100 MHz to 100 kHz. The particular radius of the frequency semicircle, *R*_ct_ and its value were calculated using the fitting program NOVA software.

### 2.6. Molecular modeling

The coordinate file of HSA (PDB code 1AO6) was retrieved from Brookhaven Protein Data Bank (http://www.rcsb.org/pdb). All non-protein atoms were manually omitted, and chain A was subjected to a 10 ns MD simulation in order to relax the side chain clashes. The 2D structure of CLO was sketched using ChemOffice 2004. Next, the 2D structure was transferred to HyperChem and the energy was optimized using the PM3 semi-empirical method. The structure with the lowest total energy was applied in docking analysis and MD simulation. The AutoDock 4.2 package was used for the docking simulation. At first, water molecules were deleted from the coordinate file of the protein. In the following step, using AutoDockTools, the missing non polar hydrogen atoms and Gasteiger charges were added to the protein structure. Finally, docking of CLO to HSA was carried out by applying AutoDock and the Lamarckian genetic algorithm.^28^

The docking procedure provides information about the binding behaviors of molecules in the binding site of a protein, which facilitates the calculation of binding energy and allows the important residues involved in the protein–ligand interactions to be evaluated. In the docking procedure, the protein flexibility is not taken into account, hence the method cannot give useful information regarding the dynamics of the binding process. Therefore, MD simulation was applied to verify the binding behavior of CLO and to obtain an insight into the overall effect of CLO on the HSA conformation. The MD simulation provides detailed information on the binding behavior of CLO including the conformational alterations in the protein structure and the stability of the CLO–HSA complex. MD simulation was carried out with the GROMOS96 force-field through the GROMACS toolkit (Ver. 5, http://www.gromacs.org).^29^ The topology file and parameters for HSA were generated by the GROMACS program. The extended single point charge (SPC/E) model was employed for water molecules.^30^ To keep the electroneutrality of the system, an appropriate number of Na^+^ counter-ions were added. The drug–protein complex was then immersed in a periodic cubic box (11.65049 × 11.65049 × 11.65049 nm^3^), and 3D periodic boundary conditions were applied to the system. The force-field parameters for CLO were computed using Automated Topology Builder (ATB) and Repository.^31^ To relieve undesirable interactions, an energy minimization process was done using the steepest descent method.^32^ The system was equilibrated over 100 ps using NVT and NPT ensembles with a temperature of 310 K set by the Nose–Hoover thermostat^33^ and a constant pressure of 1.0 bar set using the Parrinello–Rahman barostat.^34^ The long range electrostatic interactions were calculated with the Particle Mesh Ewald (PME) approach using a 1.0 nm cut off.^35^

## 3. Results and discussion

### 3.1. UV-Vis absorbance

**Fig. 1:**
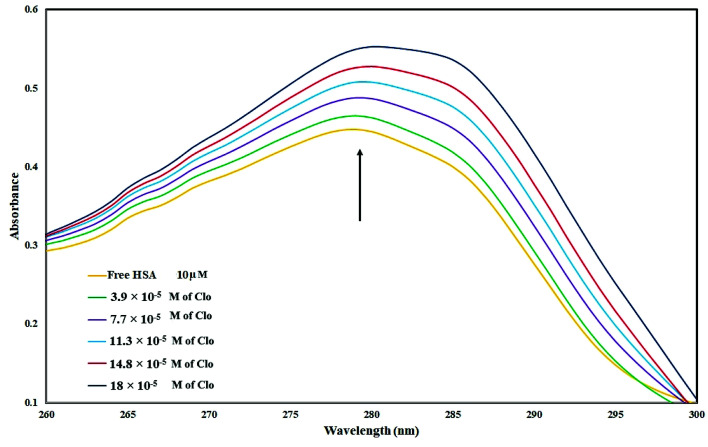
UV absorbance of HSA (10^−5^ M) with different concentrations of CLO from 3.9 × 10^−5^ to 18 × 10^−5^ mol L^−1^ at pH = 7.4. These results are clear evidence of interactions between drug and protein.

UV-Vis spectra of HSA with various amounts of CLO were recorded in order to study the structural changes of HSA during its interaction with CLO, and results are shown in Fig. 1. During the experiments, the background from CLO was eliminated by using the corresponding concentration of CLO as the reference solution. It is evident that the HSA has a specific peak at ∼280 nm. It can be seen that there are no peak shifts in the absorbance spectrum of HSA indicating no significant conformational changes in the protein structure. On the other hand, the increase in the peak intensity could be due to the binding of the drug to the excited state of the protein, which increases the absorption efficiency. These results are clear evidence of interactions between the drug and protein.

### 3.2. Analysis of fluorescence quenching

**Fig. 2:**
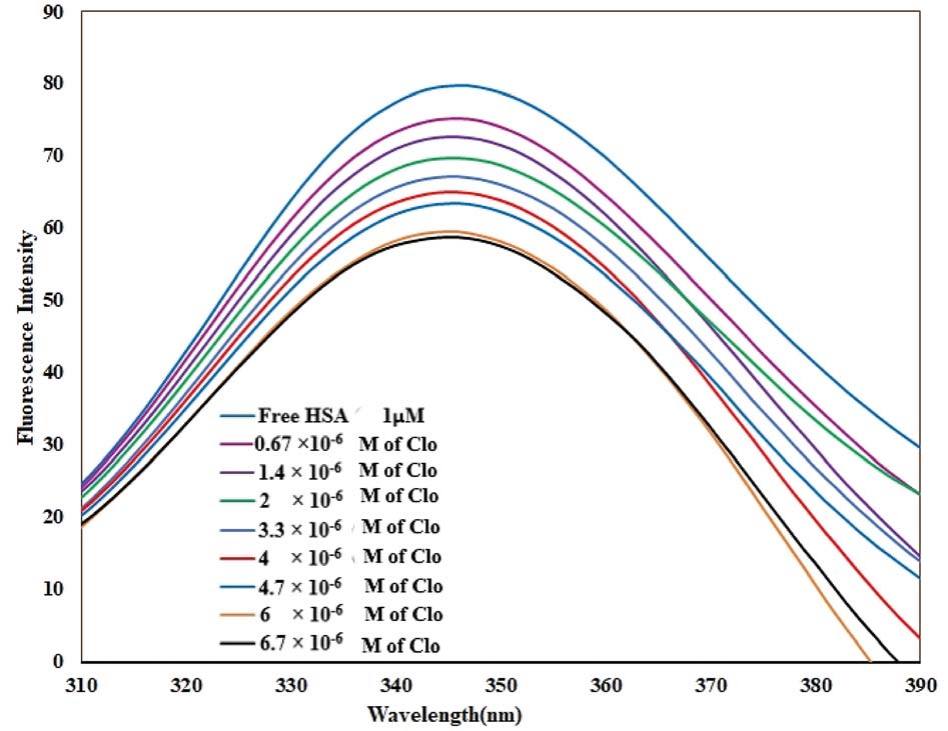
Fluorescence spectra of HSA in the absence and the presence of CLO, [HSA] = 1 × 10^−6^ M and [CLO] = 6.7 × 10^−6^ to 6.7 × 10^−7^ M, *λ*_ex_ = 289 nm, *T* = 286 K, pH = 7.4. The fluorescence intensity of the protein was reduced significantly due to quenching.

As shown in Fig. 2, when adding different concentrations of CLO to a fixed amount of HSA, the fluorescence intensity of the protein was reduced significantly due to quenching.

Quenching can happen by dynamic (collisional) and/or static mechanisms. Where the fluorophore and the quencher molecule come into contact within the lifetime of the excited state, dynamic quenching may occur. Static quenching refers to the formation of a stable complex between the quencher and the fluorophore.^36^

The fluorescence spectra were recorded at two temperatures (286 and 310 K) in order to determine the mechanism of quenching. The fluorescence quenching data were evaluated using the Stern–Volmer equation:1
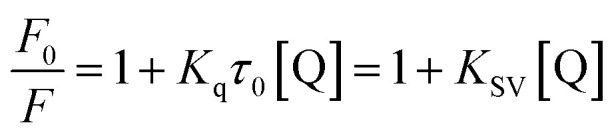
where *F*_0_ and *F* are the fluorescence intensities of HSA before and after the addition of CLO, respectively, [Q] is the concentration of CLO, *K*_SV_ is the Stern–Volmer constant, *τ*_0_ is the average lifetime of the fluorescent molecule in the absence of a quenching reagent (about 10^−8^ s), and *K*_q_ is the quenching rate constant of the biomacromolecule.^37–39^

**Fig. 3:**
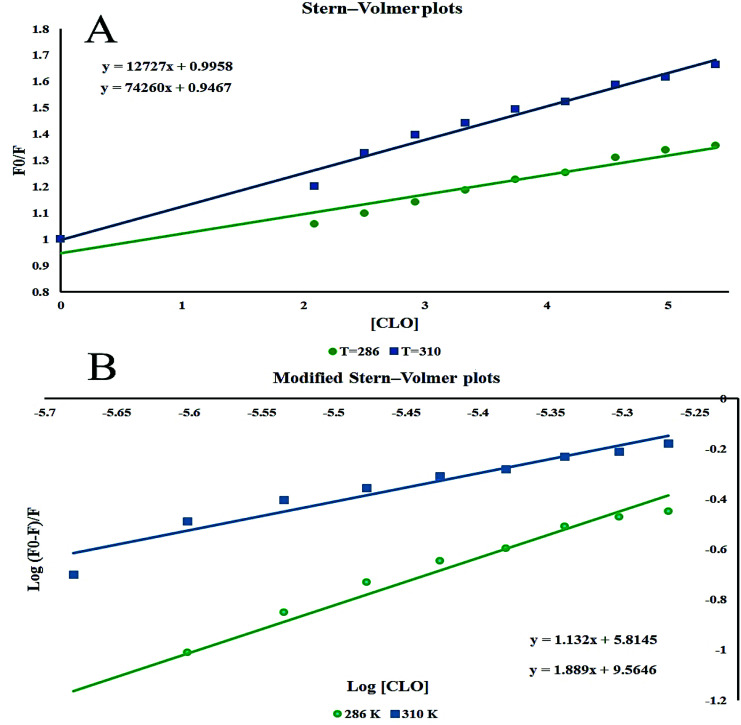
The Stern–Volmer plots at different temperatures. *λ*_ex_ = 289 nm; CHSA: 1 × 10^−6^ mol L^−1^. The slopes of the plots increase by increasing the temperature and show that a static quenching process is involved in CLO/HSA interactions.

The Stern–Volmer plots of the titrations of HSA with CLO are depicted in Fig. 3A. At the two studied temperatures, linear Stern–Volmer plots were produced with correlation coefficients of *R*^2^ = 0.94 for 286 K, and *R*^2^ = 0.98 for 310 K. The slope of the plot was greater for the higher temperature which showed that a static quenching process was involved in the CLO/HSA interactions. Furthermore, it was found that the values of *K*_q_ at both temperatures were greater than 2.0 × 10^10^ L mol^−1^ s^−1^, which also suggested that the quenching process of CLO by HSA was a static quenching process.

The binding site number (*n*) and binding constant (*K*_b_) can be determined using the modified Stern–Volmer plot (Fig. 3B) as indicated in the following formula:^40^2
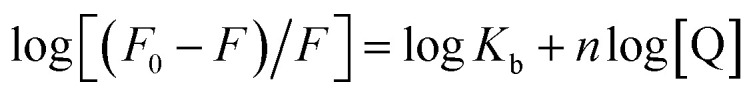


From the slope and the intercept of the plot of the log[(*F*_0_–*F*)/*F*] values *versus* log[Q] (Table 1), *K*_b_ and *n* can be derived.

**Table 1:** The Stern–Volmer constants (*K*_SV_), quenching constants (*K*_q_), binding constants (*K*_b_), number of binding sites (*n*) and relative thermodynamic parameters for the CLO–HSA system at different temperatures

*T* (K)	*K*_SV_ (L mol^−1^)	*K*_q_ (L mol^−1^)	*K*_b_ (mol L^−1^)	*n*	Δ*S* (J mol^−1^ K^−1^)	Δ*H* (kJ mol^−1^)	Δ*G* (kJ mol^−1^)
286	7.42 × 10^4^	1.23 × 10^11^	3.67 × 10^9^	1.889	−581.48	−2.18 × 10^5^	−5.2 × 10^4^
310	12.72 × 10^4^	2.12 × 10^11^	6.52 × 10^5^	1.132	−594.07		−3.4 × 10^4^

Table 1 shows the obtained values of *K*_b_ and *n* for CLO at the two different temperatures. As can be seen, with increasing temperature, the *n* value decreased.

### 3.3. Determination of free energy using the binding constant

Binding interactions of a small molecule with a macromolecule may include hydrophobic forces, electrostatic interactions, van der Waals interactions and hydrogen bonding.

Understanding the interaction of CLO with HSA requires the determination of thermodynamic parameters affected by binding, such as the changes of enthalpy (Δ*H*), entropy (Δ*S*), and free energy (Δ*G*), using the following equations:3
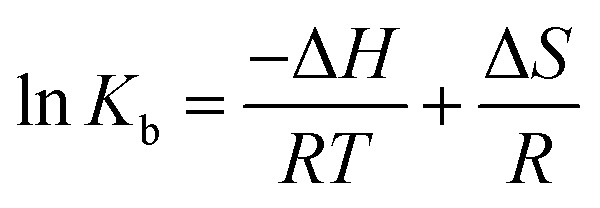


Using the slope and the *y*-intercept of the plot ln *K versus* 1/*T*, the values of Δ*H* and Δ*S* can be specified. The change at free energy with each temperature can be calculated by eqn (4):

4 Δ*G*(*T*) = Δ*H* − *T*Δ*S*

It is known that the changes in magnitude and sign of the thermodynamic parameters can be employed to determine the main interaction(s) between a biomacromolecule and a ligand.^41^ When Δ*H* > 0 and Δ*S* > 0, the main interaction forces are hydrophobic; a combination of Δ*H* < 0 and Δ*S* < 0 implies the existence of hydrogen bonds and van der Waals interactions; and the combination Δ*H* < 0 and Δ*S* > 0 indicates electrostatic interactions.^42^ In our work, the negative Δ*G* implies a spontaneous interaction process and the negative values for Δ*H* and Δ*S* indicate that hydrogen bonds and van der Waals interaction forces are involved in CLO and HSA interactions.

### 3.4. Conformational changes studied by synchronous fluorescence spectroscopy (SFS)

Fluorescence spectroscopy is a valuable technique for the study of interactions between small molecules and biomolecules. It can provide important information on the structure and the microenvironment of the fluorescent chromophore from measurements of the characteristic emission, fluorescence polarization, energy transfer and fluorescence lifetime of the biopolymer. In SFS, the sensitivity associated with general fluorescence is maintained while offering several further characteristics: narrowing of the spectral bands, simplification of emission spectra and contraction of the spectral range. Because of its sharp, narrow spectra, SFS serves as a very simple, effective method for the quantitative determination of proteins.

In order to obtain greater understanding about the binding of CLO to HSA, SFS analysis was performed. SFS involves the simultaneous scanning of the emission and the excitation monochromators of a spectrofluorimeter while maintaining a fixed wavelength difference (Δ*λ*) between them. SFS has been mainly employed to investigate the microenvironment of amino acid residues of proteins.^43^ The fluorescence signals of HSA from Δ*λ* = 15 and Δ*λ* = 60 nm are associated with the tyrosine and tryptophan residues, respectively. The fluorescence spectrum of HSA is mainly due to the Tyr and Trp residues that are sensitive to changes in their microenvironment. By investigating the synchronous fluorescence spectra of these residues, we could explore the conformational changes of HSA.

**Fig. 4:**
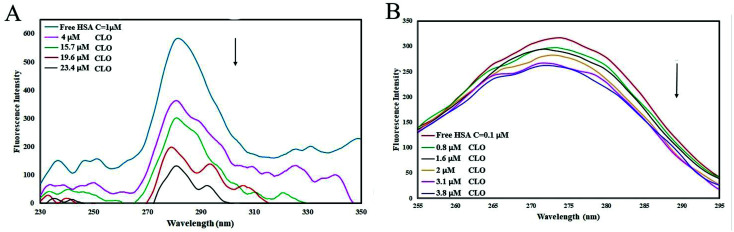
Synchronous fluorescence spectra of HSA with (A) Δ*λ* = 15 nm and (B) Δ*λ* = 60 nm in the absence and presence of increasing amounts of CLO. There are no significant changes in peak positions, indicating no significant structural changes in protein conformation.

The effect of the CLO concentration on SFS is shown in Fig. 4. The fluorescence spectra for Δ*λ* = 15 and Δ*λ* = 60 show no significant changes in peak positions, indicating no significant structural changes in protein conformation. Only a decrease in peak intensity was observed, which is due to fluorescence quenching. These results are in close agreement with the UV-Vis analysis results and both indicate that there is no significant deformation of the HSA conformation after addition of CLO. As is clear in the UV absorbance peaks, there is no right or left shift which may indicate structural stability.

### 3.5. FT-IR spectroscopy

**Fig. 5:**
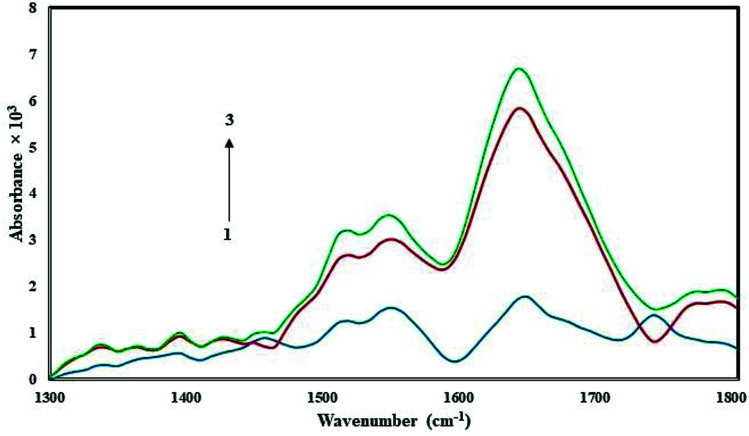
FT-IR spectra in the region of 1300–1800 cm^−1^ for 1.0 × 10^−5^ M of free HSA (blue line) and the CLO–HSA complex with ratios of 1 : 1 (red line) and 3 : 1 (green line). According to the results it can be concluded that there are no significant conformational changes in the secondary structure of HSA.

FT-IR spectroscopy is a powerful tool used to provide useful information regarding the conformational and structural details of a protein's secondary structure. Fig. 5 presents the FT-IR spectrum of free HSA in Tris–HCl buffer, and the spectra of CLO–HSA complexes. Based on the available literature, we expect protein structural changes to correspond to a spectral shift in amide I (1650 cm^−1^, mainly due to the C=O stretch) and amide II (1550 cm^−1^, the C–N stretch coupled with N–H bending) bands.^20,44,45^ An increase of the absorbance intensity for the protein amide I and amide II was observed in the spectra of the CLO–HSA complex. Both peaks of the protein are visible in both the presence and absence of CLO and they do not change in positions. Also with increasing drug concentration, no left or right shift is observed. In good agreement with the SFS and UV analyses, it can be concluded that there are no significant conformational changes in the secondary structure of HSA upon CLO binding.

### 3.6. Voltammetric studies

**Fig. 6:**
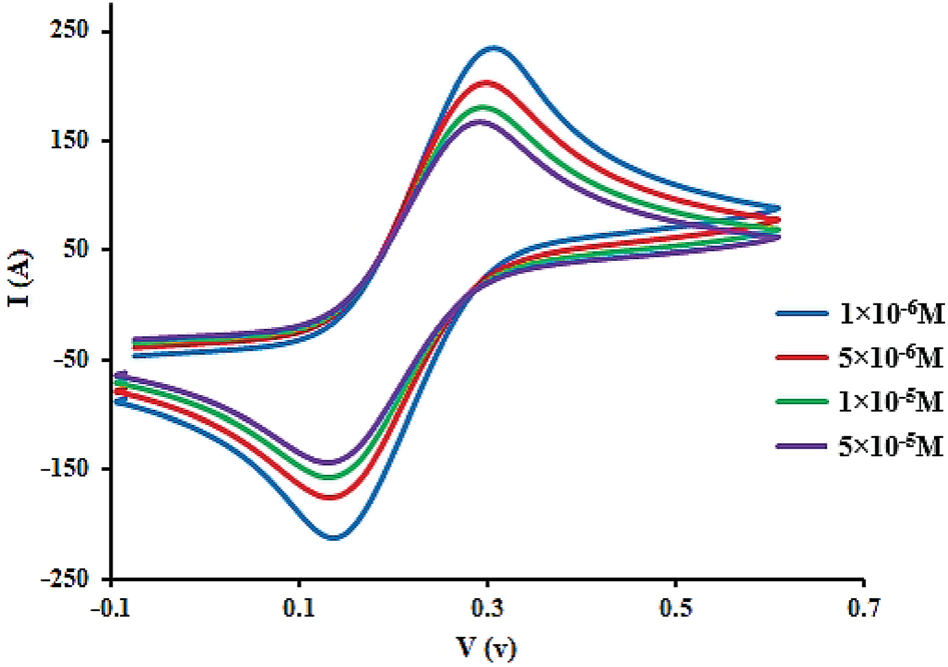
Cyclic voltammograms of CLO (1 × 10^−4^ M) at working electrode with the potential scan rate of 100 mV s^−1^ in the presence of HSA with concentrations of 1 to 50 μM. The reduction of the peak height may be assigned to the formation of a non-active electrochemical complex.

Fig. 6 displays the electrochemical behavior of CLO at the glassy carbon (GC) electrode using cyclic voltammetry. As can be seen, there is an anodic peak potential at +0.116 V and a cathodic peak potential at +0.337 V. The cyclic voltammograms of CLO at the working electrode start at ∼1.5 V and the direction is reversed at ∼0.6 V. The cyclic voltammograms of CLO (with a concentration of 1 × 10 ^−4^ M) at the working electrode in the presence of HSA are shown in Fig. 6. No new redox peak appears after adding HSA to CLO, but the anodic and cathodic peak currents are reduced. The reduction of the peak currents may be assigned to the formation of an non-active electrochemical complex.^46^ These results are consistent with the results of SFS.

Owing to the large size of the protein, it usually takes time for different drugs to reach equilibrium with HSA. For the complex of CLO–HSA, a further decrease in peak currents was observed at longer times and this remained constant after 120 s. Consequently, to obtain equilibrium conditions, any mixture of HSA and CLO was allowed to stand for 120 s before measurements were taken. The structure of the CLO has two electroactive parts, *i.e.*, the 2′- and 4′-NH_2_ groups, and both were embedded into the active site of the HSA. This binding prevents interactions at the electrode surface and therefore the contribution of these electroactive groups to the redox reaction.

**Fig. 7:**
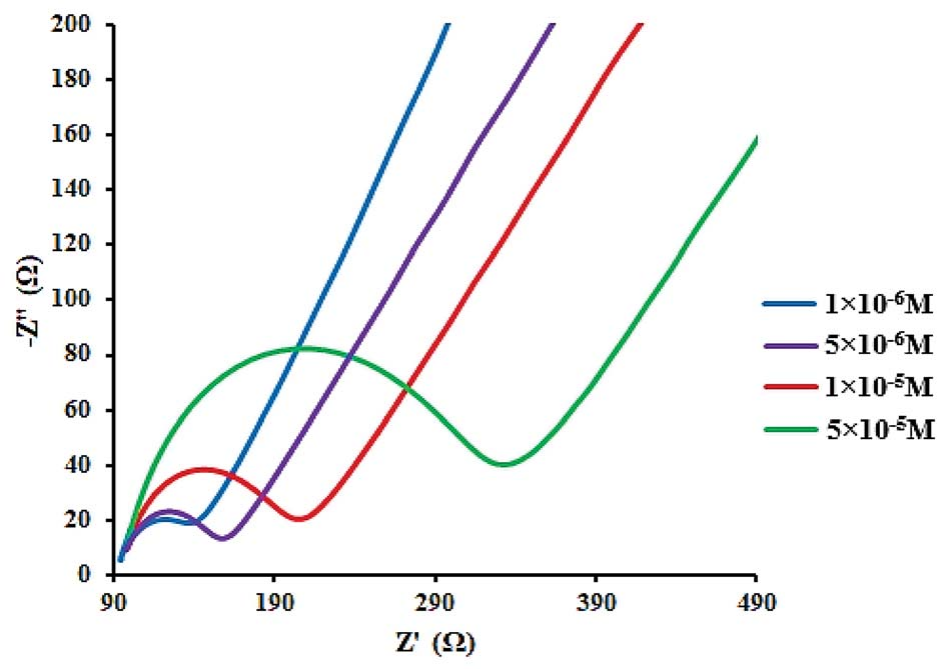
Electrochemical impedance spectra of CLO–HSAat varying concentrations of HSA (1 to 50 μM). The concentration of CLO was 10^−4^ M. This phenomenon shows CLO binds the protein through the formation of a complex on the surface of electrode causing increasing electrode resistance.

In addition, the concentration of free CLO at the electrode surface decreased within the period of the study, and hence, the peak current was further diminished. As a result of the CLO interaction with HSA, the molecular environment of CLO was changed and slight potential shifts of the reduction and oxidation peaks were observed. In another experiment, only CLO was injected into the electrochemical cell and a constant value of HSA (1.0 × 10^−4^ M) was maintained; the alteration of the electron-transfer resistance (*R*_ct_) value represented the change of CLO surface binding and blocking. From Fig. 7 and compared to CLO, *R*_ct_ increased after the addition of HSA in a dose-dependent pattern. This phenomenon shows that CLO binds to the protein through the formation of a complex on the surface of the electrode and this leads to increasing electrode resistance.

As evident from Fig. 7, after addition of CLO, the *R*_ct_ was enhanced in a dose dependent manner. In conclusion, it should be noted that the formation of a non-active electrochemical complex is consistent with the results of the cyclic voltammetry.

The binding equilibrium constant was calculated from the following equation:^47^5
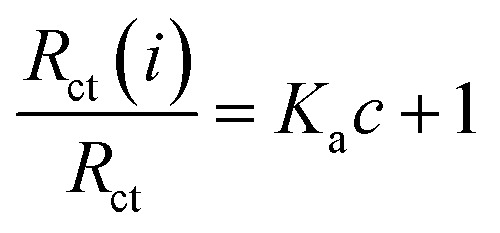
where *K*_a_ is the affinity constant for the binding of a ligand to HSA, *c* is the ligand concentration, *R*_ct_ is the electron-transfer resistance for the pure has, and *R*_ct_(*i*) refers to the electron-transfer resistance after the addition of CLO. According to the above equation, the calculated value for the affinity equilibrium constant of the CLO–HSA complex was *K*_a_ = 3.42 × 10^7^ M^−1^ at room temperature. However, the estimated affinity constant (3.42 × 10^7^ M) is relatively higher than the *K*_b_ value (3.67 × 10^5^ M) obtained from the fluorescence approach. This observed difference may be because the impedance observations could result from the binding of CLO to HSA at all of the possible binding sites leading to an enhancement of the *R*_ct_ for the studied electrode, while only the binding happening around the tryptophan residues was observed in the fluorescence experiment.^48^ Also when absorption takes place on the surface of a working electrode, the formation of the CLO–HSA complex may be different from that in the bulk of the voltammetric cell. Although the principles and descriptions of the affinity constants for the voltammetry method and fluorescence quenching differ, both of them confirmed that a static quenching mechanism is involved in the interaction between CLO and HSA.

### 3.7. Molecular docking and MD simulation results

**Fig. 8:**
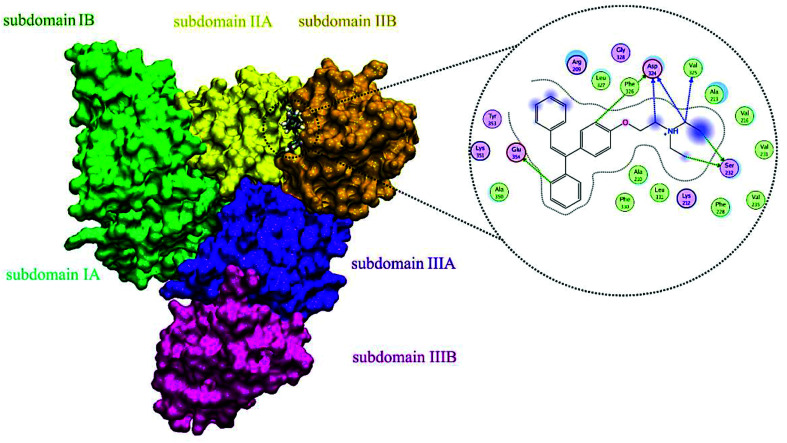
Molecular docking model of CLO located within the pocket in between subdomains IIA and IA of HSA and 2D representation of the involved residues and important interactions. The results show significant interactions between the receptor and the ligand, and determine the precise location of the interaction.

A docking approach was applied to study the major interactions in CLO–HSA and also the essential residues involved. The 3D structure of crystalline HSA shows that this polypeptide contains three homologous domains (named I, II, and III): I (residues 1–195), II (196–383) and III (384–585); and each domain has two distinct subdomains (A and B) that resemble heart shaped molecules.^19,42^ The important sites of ligand binding to HSA are located in hydrophobic cavities in the two subdomains IIA and IIIA, which correspond respectively to sites I and II. A tryptophan residue (Trp214) is located in subdomain IIA of HSA.^20^ The molecular docking results obtained using the AutoDock software confirm the results of the experimental methods. As seen in the Fig. 8, the majority of the amino acids around the drug are hydrophobic, indicating that van der Waals forces are one of the main forces involved in the interaction. Also, based on Fig. 8, it is clear that the residues of Ser232, Val325, Asp324 and Phe326 have hydrogen bonding interactions with CLO. So, the results of docking support the idea that hydrogen bonds and van der Waals interactions are involved in the ligand and protein interactions. The CLO molecule is located near Trp214, which can facilitate energy transfer and subsequent quenching.

Docking results, which show significant interactions between the receptor and the ligand, determine precisely the location of the interaction, but ultimately we need to use MD to understand the changes in the structure of the protein on interaction with the drug. These methods follow the dynamics of molecular interactions over time and show the structural and thermodynamic changes in the system during the simulation time.

**Fig. 9:**
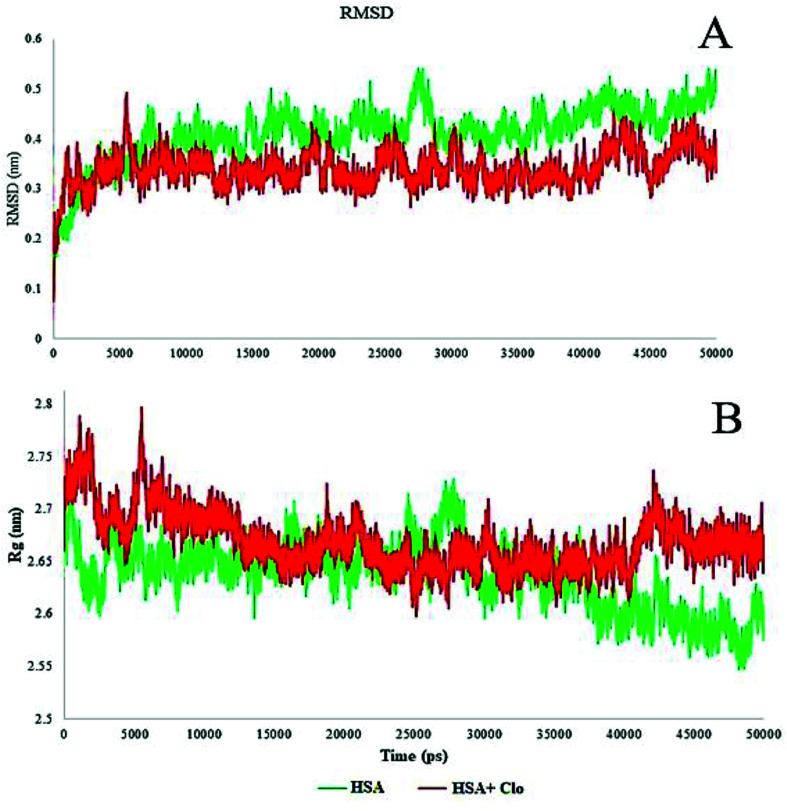
(A) Plot of root mean square deviation (RMSD) of Cα backbone *vs.* simulation time for solvated HSA and CLO–HSA. (B) Plot of radius of gyration (*R*_g_) during the MD simulation for free HSA and CLO–HSA complex. The radius of gyration indicates slight compression in the protein structure.

The MD simulation results reported the root mean square deviation (RMSD) and radius of gyration (*R*_g_) values of the protein with and without binding to the CLO in a neutralized solvated system. The RMSD values of the HSA with and without CLO were evaluated *versus* the simulation time scale (0–50 ns) and the corresponding plots are shown in Fig. 9A. The RMSD analysis indicated an initial increase due to the equilibration of the system. After 10 000 ps, the system had reached an acceptable equilibrium, there were no extreme fluctuations in the equilibrium phase and hence there were no severe structural changes. However, it must be noted that RMSD analysis cannot provide sufficient data about the conformational changes of the HSA upon binding of the ligand.

**Fig. 10:**
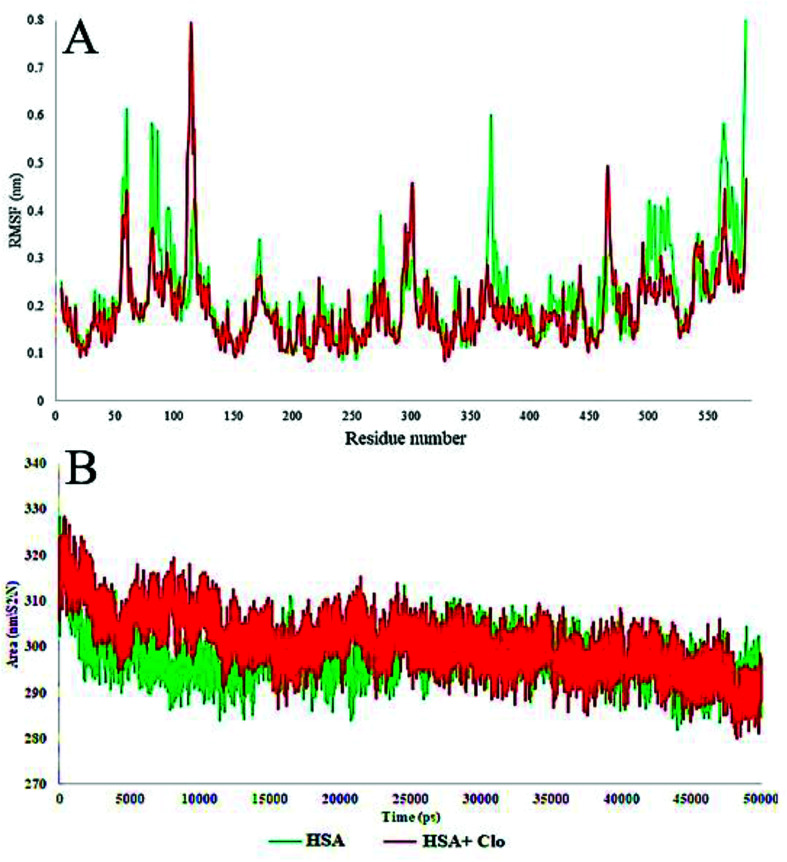
(A) Plot of root mean square fluctuations (RMSF) of Cα backbone *vs.* simulation time for solvated HSA and CLO–HSA. (B) Plot of Solvent-Accessible Surface Area (SASA) during the MD simulation for free HSA and the CLO–HSA complex. The results confirmed that after binding of CLO to HSA, the total fluctuation of the protein are reduced and the SASA calculation results indicate that CLO binding does not result in severe conformational changes.

The estimated radius of gyration (*R*_g_) values during the simulation time scale for both of the studied systems, *i.e.* free HSA and the CLO–HSA complex, are presented in Fig. 10B.

The radius of gyration analysis was performed to understand the amount of compression or protein opening that occurred in the presence and absence of drug. In the case of the free protein, initially the radius of gyration was 2.7 nm, and after the simulation time it reached 2.6 nm, indicating a slight compression in the protein structure. Compared to this state, the system containing CLO had a lower compression ratio which might be due to the drug being inhibited by greater compression. A significant reduction or increase in *R*_g_ can be a sign of a conformational change and these results further verify that there is little change, as discussed earlier with respect to the UV-Vis and FT-IR results.

The local HSA mobility per residue was investigated by calculating the time averaged root mean square fluctuation (RMSF) values of the free HSA and CLO–HSA complexes. The observed RMSF values for both systems were plotted *versus* residue numbers for all simulation times and are depicted in Fig. 10A. The difference in the levels of fluctuations for each amino acid in comparison with the mean of the total protein fluctuations was studied by using RMSF analysis. The results show that the fluctuations in the majority of the hydrophobic amino acids involved in the interaction were enhanced, while the fluctuations in the case of hydrophilic amino acids were reduced. Table 2 shows a summary of the MD analysis of HSA with CLO. The results also confirmed that after binding of CLO to HSA, the total fluctuations of the protein were reduced.

**Table 2:** MD analysis summary for HSA and CLO–HSA

Parameter	Free HSA	CLO–HSA
Mean RMSD (nm)	0.4118	0.340974
Mean RMSF (nm)	0.215776	0.194716
Mean SASA (nm^2^)	297.7111	300.9545
Mean gyration (nm)	2.63746	2.669407

The solvent-accessible surface area (SASA) defines the surface area of a group that is accessible to a solvent probe. The SASA values for free HSA and CLO–HSA were calculated and compared during MD simulation and are reported in Fig. 9B. The SASA calculation results indicate that binding of CLO results in localized conformational changes. If a severe conformational change occurred, the accessibility of the protein for solvent would significantly change, and so the SASA results again clearly verify the results of the experimental methods, especially SFS, FTIR and UV-Vis spectroscopy.

**Fig. 11:**
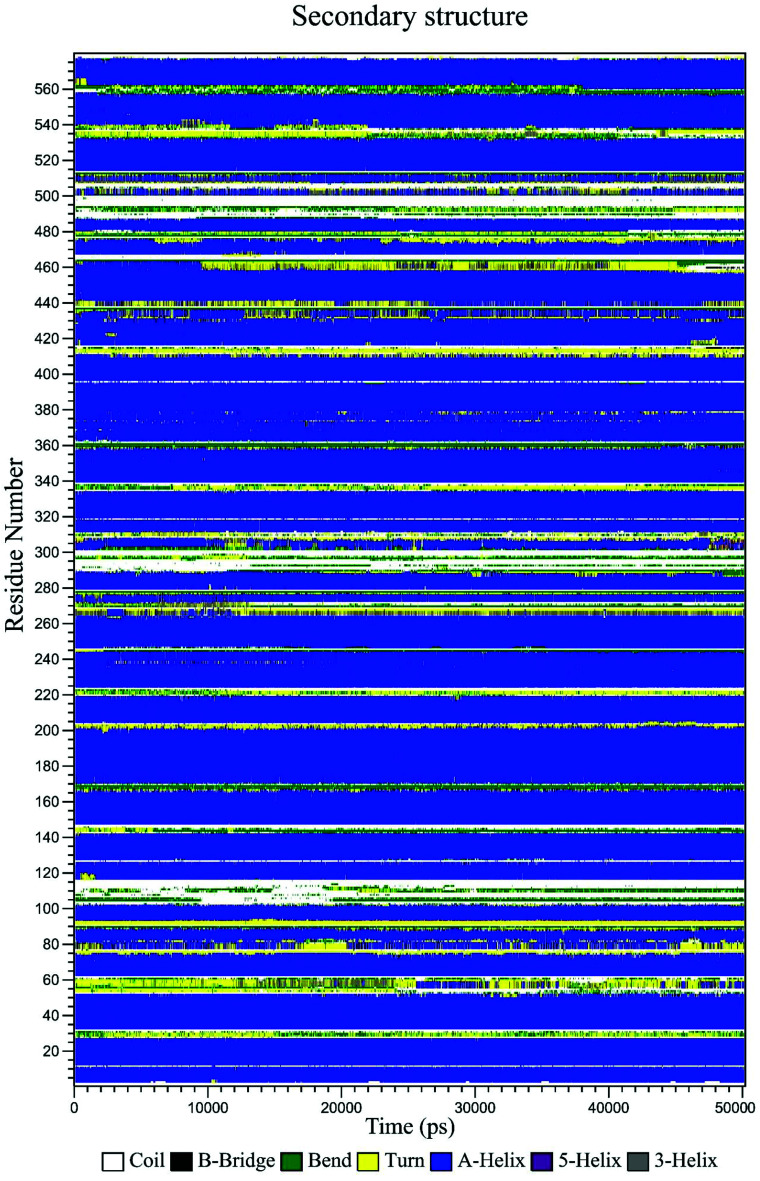
Variation of the secondary structure of HSA *versus* time for the CLO–HSA complex. The DSSP plot displays slight changes at some locations of the protein as a function of time.

The secondary structure of HSA in the CLO–HSA complex was studied according to the Define Secondary Structure of Proteins (DSSP) method which can quantitatively analyze the conformational changes. DSSP produces a plot (Fig. 11) that displays the content of α-helix, β-sheet and other secondary structures of HSA during the simulation time. The overall secondary structure pattern of CLO–HSA was maintained during the 50 ns MD simulation, although there was a slight change at some points as a function of time. Fig. 11 shows that CLO binding slightly decreased the α-helix content of HSA confirming the experimental results in this study. Altogether, the results confirm that there is protein structural stability in the HSA interaction with CLO.

**Fig. 12:**
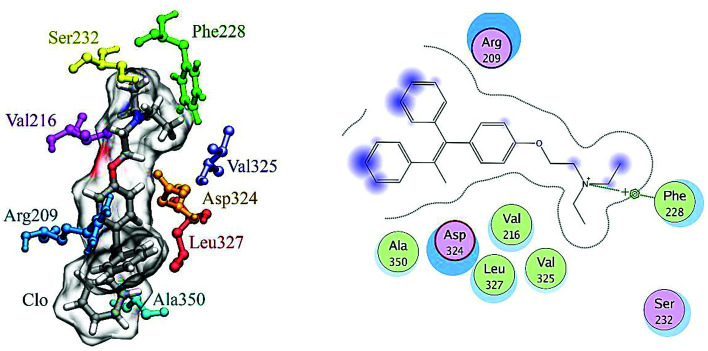
The results of MD analysis. Asp234, Phe228, Leu327 and Arg209 have the highest interaction energies in ligand-to-protein binding.

The final position of the ligand in the binding site of HSA after the MD procedure is shown in Fig. 12. The details of the energy interactions for important residues obtained using the Discovery Studio program are reported in Table 3. As can be seen, Asp234, Phe228, Leu327 and Arg209 have the highest interaction energies in ligand-to-protein binding.

**Table 3:** Interactions of HSA with CLO by residue

	van der Waals energy (kcal mol^−1^)	Electrostatic energy (kcal mol^−1^)	Interaction energy (kcal mol^−1^)
Ser232	−2.66100	1.21047	−1.45053
Phe228	−4.41375	−0.14729	−4.56104
Val216	−2.70207	1.42404	−1.27803
Ala350	1.71506	0.25735	1.9724
Leu327	−4.38351	4.13964	−0.24387
Val235	−0.58547	−0.15431	−0.739784
Arg209	−4.27911	−6.18635	−10.4655
Asp234	−4.90638	2.46302	−2.44336
All	−23.41316	3.64615	−19.767

## 4. Conclusion

An exploration of the main interactions between clomiphene (CLO) and human serum albumin (HSA) was performed by UV-Vis, fluorescence spectroscopy, voltammetry, impedance spectroscopy, FT-IR, molecular docking and MD simulation methods. The results show that there was no significant change in the fluorescence lifetime of HSA and thereby it can be concluded that the quenching of the fluorescence of HSA is mainly due to a static mechanism. The obtained negative values of Δ*H* and Δ*S* for the interactions show that hydrogen bonds and van der Waals interactions forces are involved in the binding of the CLO and protein.

A slight conformational change and changes in the secondary structure of the protein were confirmed through the FT-IR and UV-Vis spectra of HSA in the presence of the drug. From the docking analysis, it was concluded that CLO can bind in the big hydrophobic cavity of subdomain IIA of HSA, basically through van der Waals forces and hydrogen bonding to HSA. The total amount of drug in the blood is divided into two populations: the free drug and the drug bound to the protein. With an increasing tendency for the drug to bind to HSA, its clearance from the kidneys as well as its diffusion into the tissues decreases, and hence the total amount of drug in the blood increases. It is worth mentioning that the levels of free drug in the blood also decrease. It was observed that CLO interacts with HSA, and the binding affinity of CLO–HSA is fairly high, hence this may cause an excessive total accumulation of CLO in the blood along with a decrease in the levels of free drug.

## Conflicts of interest

There are no conflicts of declare.

## Supplementary Material
